# Anesthetic Management and Considerations in a Rare Case of Parietal Bone Hemangioma

**DOI:** 10.7759/cureus.60098

**Published:** 2024-05-11

**Authors:** Nicholas Koenig, Marcus Bowers, Arpan Kohli

**Affiliations:** 1 Anesthesiology, West Virginia University School of Medicine, Morgantown, USA

**Keywords:** volatile anesthetic, total intravenous anesthetic, anesthetic considerations, neuroanesthesia, parietal bone hemangiomas

## Abstract

Parietal bone hemangiomas represent a minority of diagnosed brain tumors. These lesions require careful management under anesthesia due to their vascularity and cranial location. We discuss a 31-year-old female with chronic headaches who underwent surgery for the removal of a large parietal bone hemangioma, necessitating considerations for stable hemodynamics, intracranial pressure (ICP), and bleeding risks. There is no standard anesthetic for these cases, so a mixed anesthetic approach was used, combining intravenous anesthesia with sevoflurane, aimed at optimizing control during the procedure.

## Introduction

Parietal bone hemangiomas are a class of brain tumors that make up only 0.3 to 1% of all diagnosed bone tumors [1]. They are characterized by the proliferation of vascular structures within the parietal bones [2]. The clinical presentation varies widely, ranging from incidental findings on imaging studies to symptomatic cases manifesting as headaches [1].

Magnetic resonance imaging (MRI) provides a characterization of the lesion's vascular nature, while computer tomography (CT) scans assist in the evaluation of bony changes [2]. Utilization of both imaging modalities facilitates diagnosis and determination of clinical management. The first line of treatment reported in literature reviews and case reports is surgical removal [2]. Presurgical planning is important as many of these masses are prone to bleeding and are located in regions where cosmetic deformities can occur [2,3]. For tumors that are inoperable, radiotherapy can be used; however, there is an increased risk of carcinogenesis [4].

Anesthetic management for these patients depends on the size and location of the tumor. Large hemangiomas can potentially elevate intracranial pressure (ICP) due to their vascularity and space-occupying nature [5]. Anesthetic management should focus on maintaining stable hemodynamics, avoiding factors that could increase ICP, and ensuring proper cerebral perfusion [5]. Monitoring ICP during the procedure may be considered, especially if the lesion size and location pose a risk of impinging on vital structures [5]. Another consideration during anesthesia is the potential for bleeding. In one case report, controlled hypotension was a method used to help limit bleeding during surgical resection of cavernous sinus hemangiomas [6]. Adequate preoperative assessment, blood type and crossmatching, and preparation for potential blood transfusions are essential.

In this case report, we will discuss a patient who underwent general anesthesia for the removal of a growing parietal bone hemangioma.

## Case presentation

A 31-year-old female with a past medical history of type 2 diabetes mellitus, hyperlipidemia, and a 13-year history of headaches initially presented to the neurological surgery clinic for referral after imaging findings showed two expansile T1 and T2 hyperintense parietal bone lesions.

Her headache pattern was as follows: they began in December 2020 and occurred approximately seven times per week. The headaches started in the occipital region of her head and radiated forward. The pain was described as a sharp, pounding type of pain that was severe in intensity. The associated symptoms include aggravation by movement, nausea, photophobia, and phonophobia. However, the patient did not experience an aura. In June 2021, her typical headache pattern acutely changed and worsened. The patient reported a new onset numbness/tingling and tenderness to palpation on the left temporal region of her head. In May 2022, she developed a left-sided migraine that did not completely resolve until August 2022. During that time, the patient reported a history of short-term memory issues as well. The patient trialed amitriptyline 50 mg once daily, gabapentin 300 mg once daily, carbamazepine 100 mg twice daily for prevention of the headaches, cyclobenzaprine 10 mg as needed, and acetaminophen and nonsteroidal anti-inflammatory (NSAIDs) drugs as abortive treatment with all treatments providing little relief. The patient was placed on topiramate for preventative treatment and naratriptan for abortive treatment. This resulted in better control of the frequency and severity of her symptoms. Physical examination, including a full neurological physical exam, throughout all clinic visits, was completely normal. 

**Figure 1:**
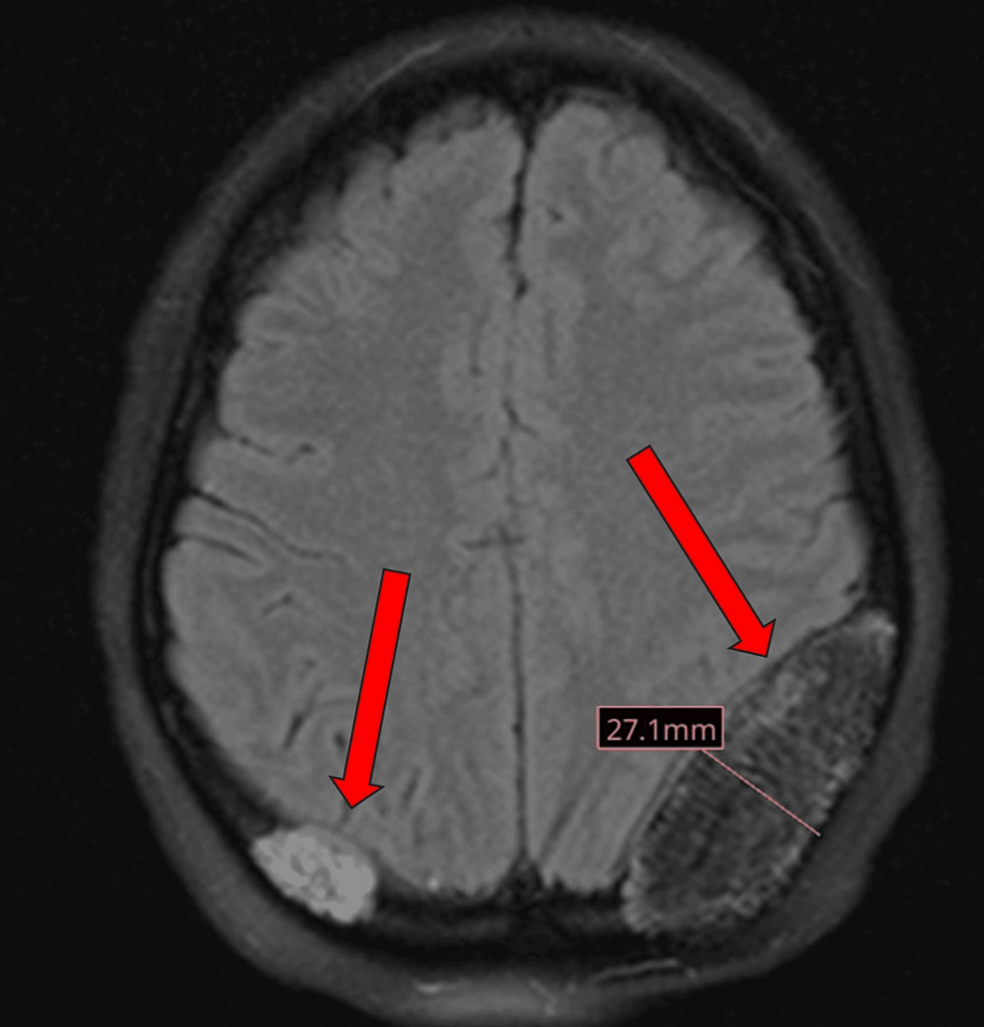
MRI brain T2 axial flair (27.1 mm) This is the initial imaging for the patient's brain masses found on MRI, suggestive of parietal bone hemangioma. MRI, magnetic resonance imaging

**Figure 2:**
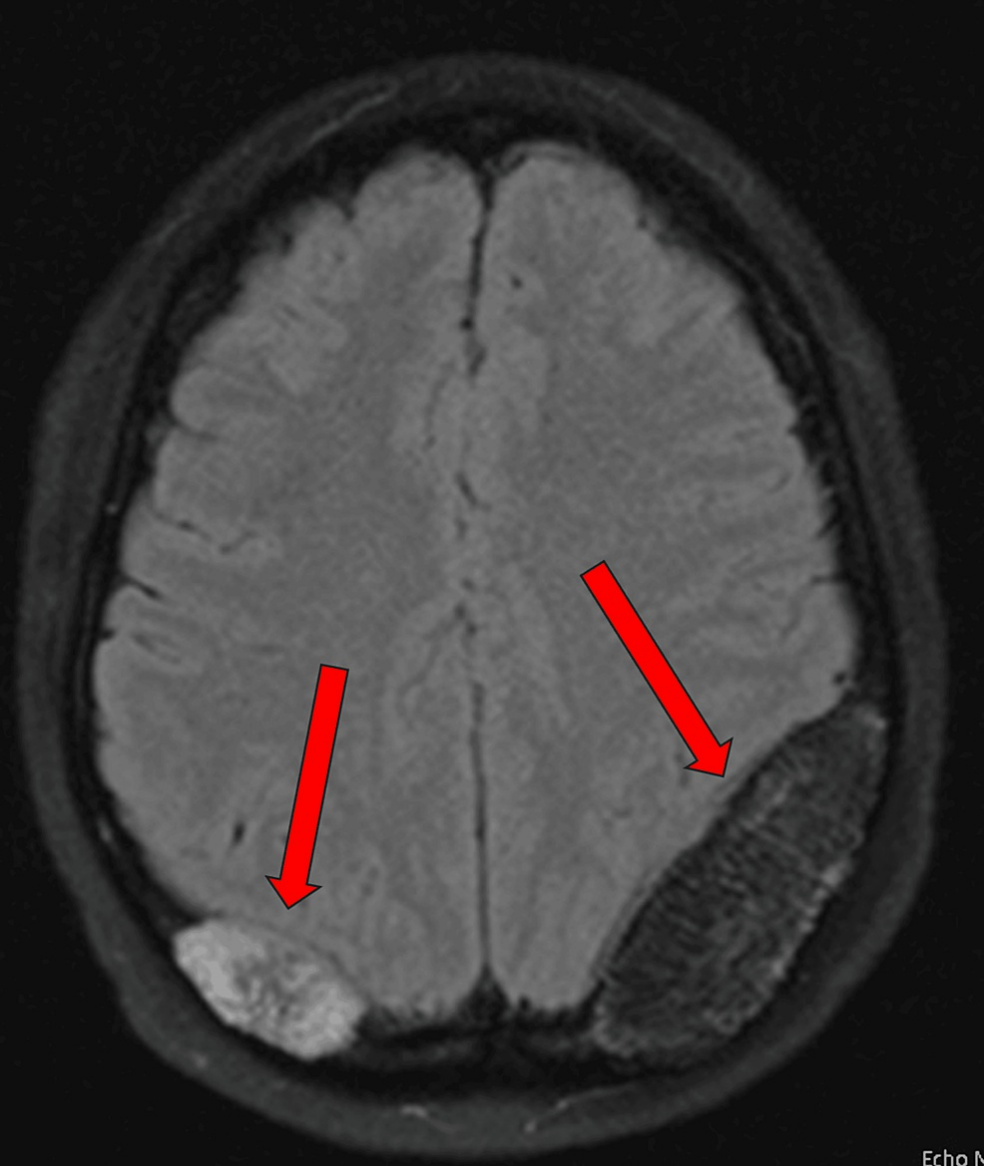
MRI brain T2 axial flair This is the follow-up imaging for the patient's brain masses found on MRI, showing growth of the right-sided lesion with the stable left-side lesion. MRI, magnetic resonance imaging

The patient had a series of MRI brain scans from May 2022 to September 2023. The initial MRI brain scan in May 2022 revealed two expansile T1 and T2 hyperintense left- and right-sided parietal bone lesions, with the lesion on the left measuring 1.8 cm and the lesion on the right measuring 1.6 cm (Figure 1). The left parietal bone mass resulted in a local mass effect on the left parietal lobe with effacement of the sulci and effacement of the left occipital horn of the lateral ventricle. An MRI brain scan in September 2023 showed no further growth of the left-sided parietal bone lesion; however, the right-sided lesion grew to 3.1 cm (Figure 2). 

In November 2023, the patient underwent a procedure for the removal of the right-sided parietal lesion due to its increasing size. During the pre-anesthetic evaluation in October 2023, the patient's medication regimen included amitriptyline 50 mg once daily, pregabalin 75 mg once daily, tizanidine 4 mg, topiramate 100 mg twice daily, metformin 1000 mg twice daily, and sitagliptin 100 mg once daily. The patient's allergies included penicillin. This patient was classified according to the American Society of Anesthesiologists (ASA) as an ASA physical status class 3. Prior to the procedure, the risk of blood loss being a concern was discussed with the patient, and consent was obtained for the use of blood products in case there was a need for a transfusion. The type and screening of the patient's blood were investigated preoperatively. The anesthetic management of this case consisted of standard induction with 2 mg midazolam, 100 mcg fentanyl, 80 mg lidocaine 2%, 200 mg propofol, and 70 mg rocuronium. Induction was uneventful. A right radial arterial line was placed post-induction. During the procedure, a one-time dose of vancomycin was given as antibiotic prophylaxis due to the patient's penicillin allergy. Anesthetic maintenance throughout the procedure was achieved with sevoflurane, 75 mcg/kg/minute propofol drip, and 0.1 mcg/kg/minute remifentanil infusion. Throughout the procedure, the patient's vitals remained stable, only requiring 400 mcg of phenylephrine to support low blood pressure. No neuromonitoring was utilized during this procedure. Emergence and extubation were uneventful and the patient was stable post-procedure in the Post-Anesthesia Care Unit (PACU). Blood loss was reported to be less than 50 mL throughout the procedure. 

## Discussion

Intraosseous cavernous hemangiomas make up a rare entity of brain tumors, comprising up to 1% of all bone tumors. The majority of these tumors are found in the spinal cord, while a minority are found in the skull [1]. In one systematic review of the literature, parietal bone hemangiomas made up 33% of the reported cases of intraosseous cavernous hemangiomas, which was comparable to frontal bone hemangiomas [2]. The diagnostic challenges associated with intraosseous cavernous hemangiomas lie in their predominantly asymptomatic nature and nonspecific clinical manifestations when symptomatic events occur [2]. In the reported case, the patient developed new-onset numbness/tingling, tenderness to palpation on the left side of the skull, and short-term memory loss, which prompted further investigation of her symptoms. Imaging, particularly MRI, played a pivotal role in achieving an accurate diagnosis. CT scans are also used in diagnostics and typically reveal characteristic expansive lesions with thin borders [1]. MRI typically shows hyperintense T2 sequences, as was the case in this patient [2]. However, it is crucial to acknowledge that these imaging features, although helpful, are not entirely specific to intraosseous cavernous hemangiomas, necessitating a comprehensive approach to differentiate them from other cranial lesions.

Surgical resection emerges as the primary treatment modality for intraosseous cavernous hemangiomas, especially in symptomatic cases with low rates of recurrence [2]. The reported case aligns with existing literature advocating for complete tumor removal. 

The anesthetic management of this case reflects one of the multiple methods of standard anesthesia for a neurosurgical procedure. Arterial line placement allowed for constant monitoring of blood pressure during a procedure where blood loss was at a higher risk. Although ICP was not measured during this procedure, it is still an important consideration for larger hemangiomas, especially with the use of volatile anesthetics due to the tendency to increase ICP above 1.0 minimal alveolar concentration (MAC) [7]. Careful fluid management is essential to prevent fluctuations in intravascular volume, which could impact bleeding and hemodynamics [8]. 

Regarding the choice between a total intravenous anesthetic (TIVA) and a volatile anesthetic, TIVA allows for better control of the depth of anesthesia and analgesia [7]. Intravenous anesthetics result in a reduction in cerebral blood flow (CBF) and therefore do not increase ICP [9]. They also cause a reduction in the cerebral metabolic rate of oxygen (CMRO2) [9]. This approach is often favored in neurosurgical procedures and in cases where controlled hypotension is necessary or when there are concerns about the effects of increased ICP or neuromonitoring from the use of volatile anesthetics [7,8]. 

Volatile anesthetics increase ICP, mainly above a MAC of 1.0, by causing an increase in CBF [9]. Volatile agents produce a dose-dependent reduction of CMRO2. Because of their intrinsic vasodilatory properties (isoflurane = desflurane > sevoflurane), volatile agents uncouple CMRO2 and CBF. Their overall effect on CBF will result in a balance between CMRO2-related CBF reduction and intrinsic vasodilatory-related increase in CBF [9]. Therefore, inhaled anesthetics such as sevoflurane and desflurane are also commonly used solely or as part of a balanced anesthetic in neurosurgical procedures. They offer neuroprotective effects and are titratable for depth of anesthesia [8]. 

Another consideration is the interference of volatile anesthetics on neuromonitoring. This involves monitoring the function of the nervous system in real time, which can include techniques like somatosensory-evoked potentials (SSEPs) and motor-evoked potentials (MEPs) [7]. Evoked potentials are decreased in amplitude, the latency of all cortical SSEPs is increased, and MEPs are significantly decreased with the use of volatile anesthetics. Neuromonitoring was not utilized during this procedure; however, depending on the lesion's location, proximity to critical neural structures may necessitate neuromonitoring during surgery. 

The anesthetic management of this case consisted of a combination of both sevoflurane and an intravenous anesthetic consisting of remifentanil and propofol. This allowed for more precise control over drug delivery and minimized the use of the inhaled anesthetic. As the decision between TIVA and volatile anesthesia is often influenced by factors such as the surgeon's preferences, institutional practices, and the patient's overall medical condition, collaborative discussions between the anesthesia and surgical teams are crucial to tailor the anesthetic approach to the individual needs of the patient and the intricacies of the surgical procedure.

## Conclusions

This case report reviews the anesthetic management of parietal bone hemangiomas. Surgical resection remains the standard treatment, with careful consideration given to preoperative planning. Although there is no standard anesthetic for these cases, proper anesthetic management is important for ensuring patient safety during surgical procedures, with a focus on maintaining stable hemodynamics, avoiding factors that could increase ICP, and ensuring adequate cerebral perfusion. The choice between TIVA and volatile anesthesia should be tailored to individual patient factors and surgical requirements. Successful outcomes can be achieved while minimizing perioperative complications in patients undergoing surgical resection of parietal bone hemangiomas with these anesthetic considerations.
